# Does the vaginal wall become thinner as prolapse grade increases?

**DOI:** 10.1007/s00192-016-3150-1

**Published:** 2016-09-27

**Authors:** Rhiannon Bray, Alex Derpapas, Ruwan Fernando, Vik Khullar, Demetri C. Panayi

**Affiliations:** 1 Department of Urogynaecology, St Mary’s Hospital, Imperial NHS Trust, London, W2 1NY UK; 2 Department of Urogynaecology, Pelvic Floor and Childbirth Injury, Epsom and St. Helier NHS Trust, Carshalton, UK

**Keywords:** Prolapse, Ultrasonography, Vaginal wall thickness

## Abstract

**Introduction and hypothesis:**

The pathophysiology of prolapse is not well understood. However, two main theories predominate: either the fibromuscular layer of the vagina develops a defect/tears away from its supports, or its tissues are stretched and attenuated. The aim of this study was to assess how vaginal wall thickness (VWT) is related to vaginal prolapse.

**Methods:**

The study group comprised 243 women with symptomatic prolapse recruited from the Outpatient Department of a tertiary referral centre for urogynaecology. A history was taken and women were examined to determine their POP-Q score. Using a previously validated technique, ultrasonography was used to measure the mean VWT at three anatomical sites on the anterior and posterior walls. Scores were then compared using *t* tests, the Kruskal-Wallis test and the Friedman test.

**Results:**

The mean age of the patients was 59.7 years (SD 12.0 years range 38 – 84 years). For each measurement VWT reduced as prolapse grade increased until the prolapse extended beyond the hymen. Women with grade 3 prolapse had a significantly higher mean VWT than women with grade 1 or 2 contained prolapse. Menopause status did not have a significant effect on the VWT.

**Conclusions:**

VWT is lower in women with vaginal prolapse until the prolapse extends beyond the hymen and then VWT is thicker and comparable with women without prolapse. This may be explained by changes in the vaginal tissue including reduction of collagen, elastin and smooth muscle, as well as fibrosis in exposed tissues, rather than by defects in the vagina.

## Introduction

Pelvic organ prolapse (POP) is a common condition, defined as the descent of the pelvic organs (bladder, urethra, vagina, uterus, small bowel or rectum) secondary to deficiencies in the pelvic support system [1]. The exact prevalence is difficult to define but current estimates suggest that a woman’s life-time risk for prolapse surgery is as high as 20 % by the age of 80 years [2]. Despite the high prevalence and significant impact on quality of life, little is known about the pathophysiology of prolapse [2–5]. It is understood to be multifactorial with age, parity, weight, collagen weakness, and molecular and genetic influences all contributing [6]. On an anatomical level, two main causative theories predominate. The defect theory proposes that discrete tears in the fibromuscular wall of the vagina or detachments from their adjacent support tissues lead to POP [7, 8]. This has been contested by an alternative theory that the vaginal wall and its supports are stretched or attenuated [9]. These two theories could be tested by measuring vaginal wall thickness (VWT) and having consistency of thickness.

The vaginal wall itself is composed of three layers: epithelium, muscularis and adventitia [10]. The epithelium is nonkeratinized, nonsecretory, stratified squamous epithelium, the muscularis is primarily smooth muscle with small amounts of collagen and elastin, and the outer adventitia is a fascial layer of connective tissue which includes collagen, elastin, adipose tissue, blood vessels and nerves. The fascia can be further divided into two types: the parietal fascia which covers the muscles and whose attachments to the vagina are referred to as endopelvic fascia, and the visceral fascia which covers the organs [11]. Anteriorly the vaginal wall is related to the base of the bladder and the urethra, and the fascia is referred to as the pubocervical fascia, and posteriorly the rectovaginal fascia separates the vagina from the rectum and the perineal body [12]. Little is known about the response of the vaginal wall to the pressure of prolapse, but weakening of these tissues is thought to be a contributing factor. The attenuation theory suggests that the tissues become thinner as they are stretched; this would not be expected in the defect theory [12].

Transvaginal ultrasonography has long been used for measurements within the pelvis [13]. Our technique for the measurement of VWT is highly reproducible and has been validated against the gold standard of histology [14]. The measurement can be taken in real time conferring an advantage over other imaging modalities and histopathological assessment. To further our understanding of vaginal prolapse, the aim of this study was to assess the relationship between VWT and vaginal prolapse grade.

## Materials and methods

**Fig. 1:**
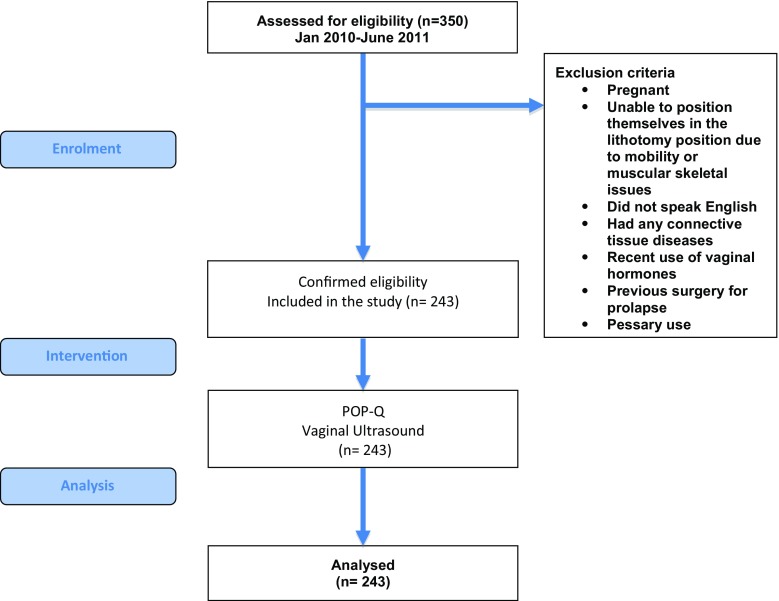
Study flow chart showing exclusion criteria, intervention and analysis

Women with symptomatic prolapse were recruited over an 18-month period from the Outpatient Department of a tertiary referral centre for urogynaecology (Fig. 1). Women were excluded if pregnant, were unable to position themselves in the lithotomy position due to mobility or muscular skeletal issues, did not speak English or had any connective tissue diseases, or recent use of vaginal hormones, or previous surgery for prolapse or pessary use. Institutional Review Board approval was obtained prior to the start of the study, and all patients signed an informed consent form prior to inclusion.

**Fig. 2:**
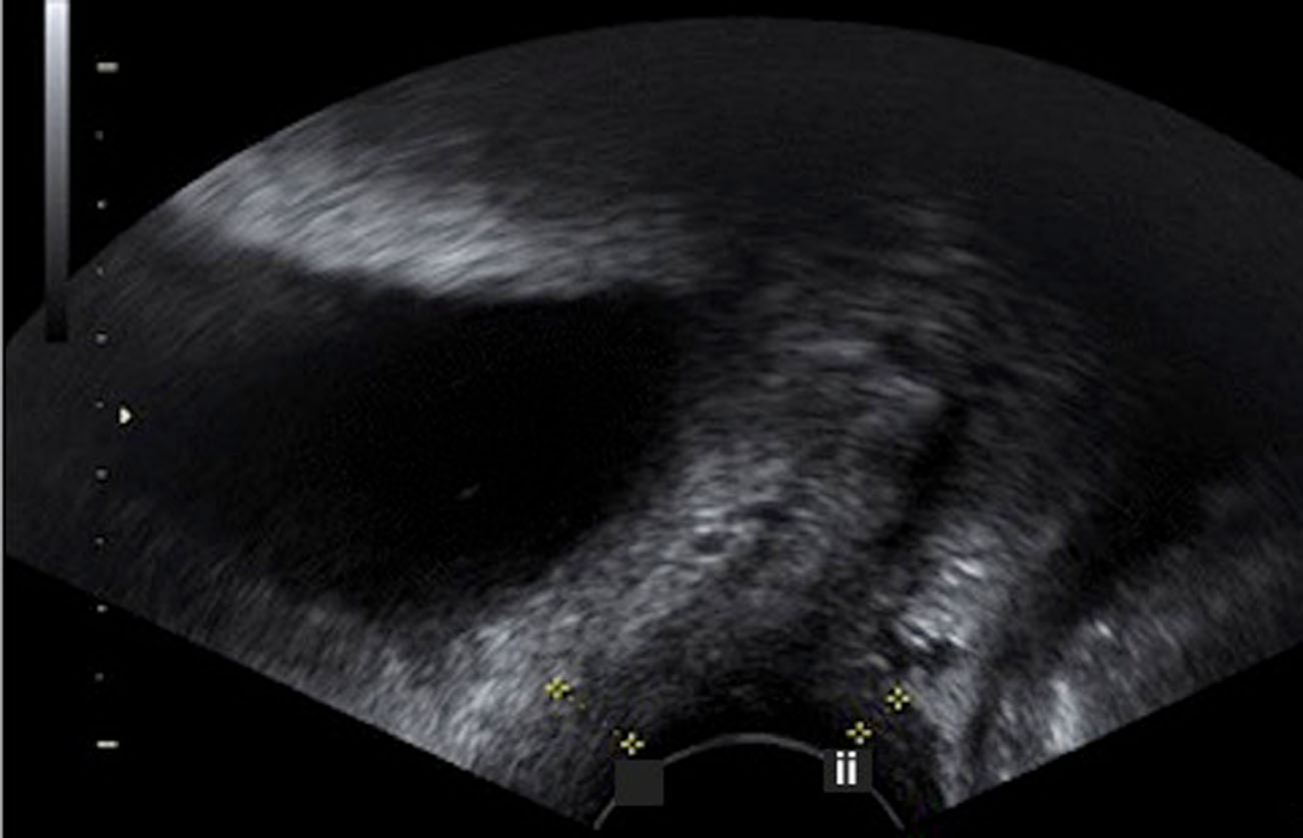
Ultrasound image showing measurement of vaginal wall thickness at the level of the bladder neck *i* and the anorectal junction *ii*

Women were examined to determine their POP-Q score and ultrasonography was performed using a previously validated technique [14]. Women were scanned in the lithotomy position whilst performing a Valsalva manoeuvre, within 15 min of emptying their bladder and on confirming by ultrasonography that their bladder contained less than 50 ml of urine. VWT was determined at the bladder neck (VWTBN), at the level of the dome of the bladder (VWTB), and at the anterior fornix (VWTAF), and at the level of the anorectal junction (VWTARJ), the rectum (VWTR) and the posterior fornix (VWTPF), posteriorly. VWT included the full thickness of tissue between the vaginal lumen and the prolapsed pelvic organ (Fig. 2). It was not possible to blind the sonographer to the POP-Q score prior to the ultrasound scan.

Women were grouped according to the severity of prolapse. In women with a POP-Q score of less than −2, the prolapse was considered grade 0, in women with a score of −2 to −1 as grade 1, in women with a score of −1 to 0 as grade 2, and in women with a score greater than 1 as grade 3. Scores were compared to VWT at the three anatomical sites on the anterior and posterior vaginal walls. The primary outcome measure was the relationship between mean VWT and prolapse grade. Secondary outcome measures were the relationship between mean VWT at each of the points anteriorly or posteriorly for different grades of prolapse, and the effect of menopause status on VWT.

Statistical analysis was performed with SPSS Statistics v. 23 (IBM Corp., Armonk, NY). Parametric data were analysed using *t* tests and nonparametric data were analysed using the Kruskal-Wallis test.

## Results

**Fig. 3:**
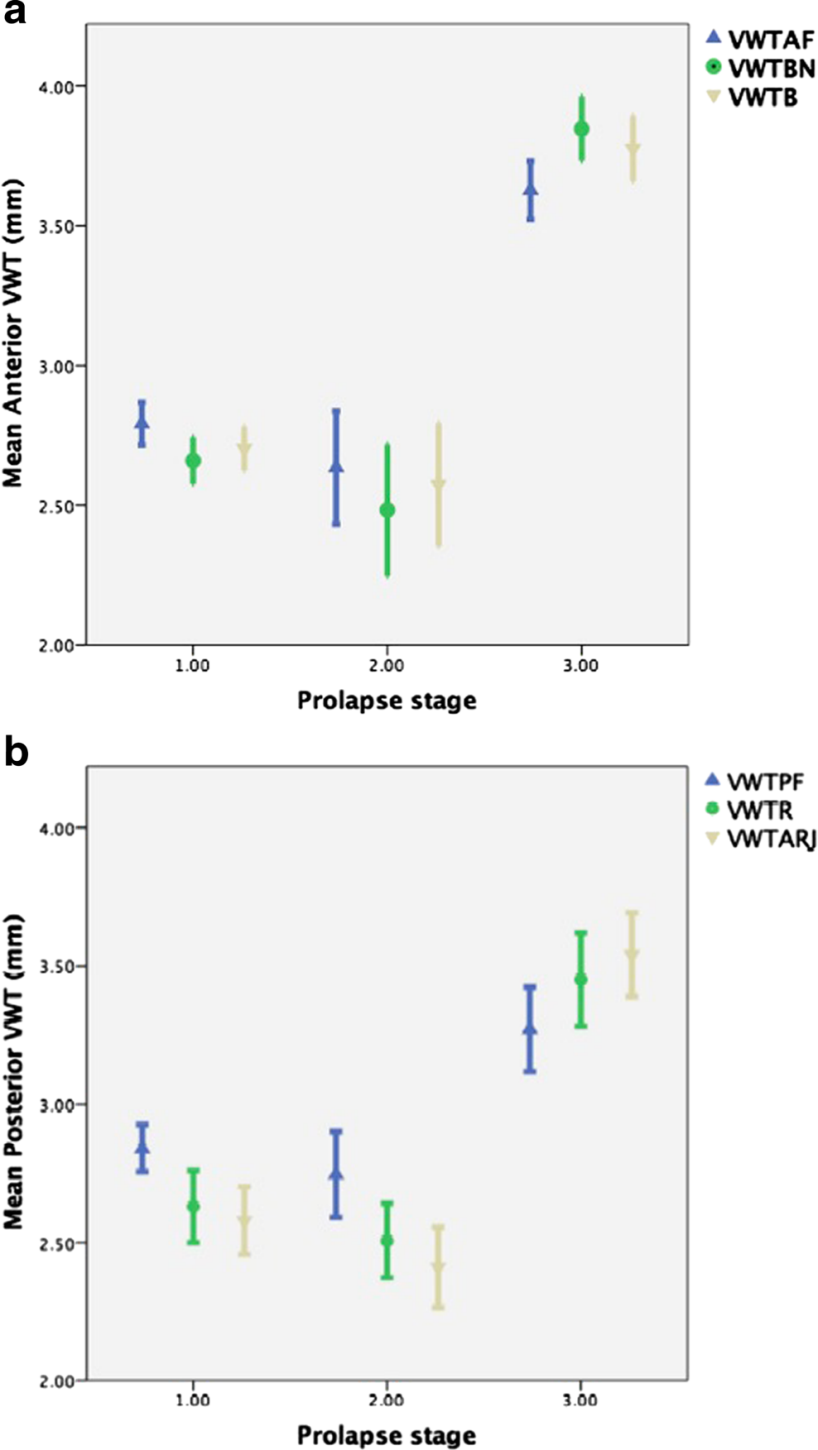
Vaginal wall thickness (*VWT*) in relation to prolapse grade. **a** Anterior compartment with prolapse graded according to POP-Q score at point Aa (*p* < 0.001): fornix (*VWTAF*), bladder neck (*VWTBN*), and bladder (*VWTB*). **b** Posterior compartment with prolapse graded according to POP-Q score at point Ap (*p* < 0.001): anorectal junction (*VWTARJ*), rectum (*VWTB*), and fornix (*VWTPF*). The data are presented as means with error bars indicating 95 % confidence intervals

A total of 243 women were recruited. Their mean age was 59.7 years (SD 12.0 years, range 38 – 84 years), and their average grades of prolapse were 1 in the anterior compartment, 0 in the central compartment and 1 in the posterior compartment 1 (Table 1). The mean VWT at each point on the anterior and posterior vaginal walls for each grade of prolapse are shown in Fig. 3 and Table 2. For each point on the anterior and posterior vaginal walls, the mean VWT reduced as prolapse grade increased until the prolapse extended beyond the hymen (*p* < 0.001; Fig. 3, Table 2). In grades 1 and 2 prolapse, anteriorly the vaginal wall was thickest at the anterior fornix, followed by the bladder, followed by the bladder neck. This pattern was reversed in patients with grade 3 prolapse. The same pattern was also observed in the posterior vaginal wall (*p* < 0.05; Table 2). In both the anterior and posterior compartments, VWT at each point was significantly higher in grade 3 prolapse than in grades 1 and 2 prolapse combined (*p* < 0.001, Kruskal Wallis; Table 3).

**Table 1:** Numbers of patients with each grade of prolapse

Grade of prolapse	Anterior compartment (*n*)	Posterior compartment (*n*)	Central compartment (*n*)
0	0	0	182
1	164	163	14
2	23	33	17
3	55	45	22

The median grade of prolapse in the anterior compartment was 1, in the posterior compartment was 1, and central compartment was 0

**Table 2:** Vaginal wall thickness at each of the six points. Mean and 95 % CI shown

Measurement point	Vaginal wall thickness (mm)						*p* value (Kruskal-Wallis test)
	Prolapse grade 1		Prolapse grade 2		Prolapse grade 3		
	Mean	95 % confidence interval	Mean	95 % confidence interval	Mean	95 % confidence interval	
Anterior compartment							
Bladder neck	2.66	2.58 – 2.74	2.48	2.25 – 2.72	3.85	3.73 – 3.96	<0.0001
Bladder	2.70	2.62 – 2.78	2.57	2.35 – 2.79	3.78	3.66 – 3.89	<0.0001
Fornix	2.79	2.72 – 2.87	2.63	2.43 – 2.83	3.63	3.52 – 3.73	<0.0001
*p* value (Friedman test)	<0.0001		<0.029		<0.0001		
Posterior compartment							
Anorectal junction	2.58	2.46 – 2.70	2.41	2.26 – 2.56	3.54	3.39 – 3.69	<0.0001
Rectum	2.63	2.50 – 2.76	2.51	2.37 – 2.64	3.54	3.39 – 3.69	<0.0001
Fornix	2.84	2.76 – 2.92	2.75	2.59 – 2.90	3.27	3.12 – 3.42	<0.0001
*p* value (Friedman test)	<0.0001		<0.0001		<0.0001		

Measurements at each point are significantly different between grades of prolapse

**Table 3:** Vaginal wall thickness at each measurement point comparing prolapse grades 1 + 2 and prolapse grade 3

Measurement point	Vaginal wall thickness (mean, mm)		*p* value (Kruskal-Wallis test)
	Grades 1 + 2	Grade 3	
Bladder neck	2.64	3.85	<0.001
Bladder	2.69	3.78	<0.001
Anterior fornix	2.77	3.63	<0.001
Anorectal junction	2.56	3.06	<0.001
Rectum	2.61	3.05	<0.001
Posterior fornix	2.79	3.05	<0.001

Menopause status did not have a significant effect on VWT (Table 4). We did not compare the anterior and posterior measurements as the forces and organs involved are different.

**Table 4:** Effect of menopause status on vaginal wall thickness

Measurement point	Vaginal wall thickness (mean, mm)		*p* value (*t* test)
	Premenopausal (*n* = 65)	Postmenopausal (*n* = 178)	
Bladder neck	2.90	2.91	0.56
Bladder	2.90	2.94	0.79
Anterior fornix	2.96	2.97	0.65
Anorectal junction	2.75	2.71	0.91
Rectum	2.75	2.75	0.77
Posterior fornix	2.77	2.91	0.17

## Discussion

Our results show that VWT is related to the grade of vaginal prolapse. For prolapses that have not descended further than the hymen, VWT decreases with increasing grade, and for those that extend beyond the hymen, VWT increases. We also observed that VWT was significantly less in women with grade 1 or 2 prolapse combined than in those with grade 3 prolapse. VWT increased caudally in women with grade 1 or 2 prolapse, and decreased caudally in women with grade 3 prolapse.

No studies have examined VWT in patients with different grades of prolapse. However, in line with our results, a study comparing VWT in premenopausal and postmenopausal women with grade 1 or 2 prolapse showed a significantly greater epithelial thickness in the proximal segment of the posterior wall than in the distal segment [15]. Hsu et al. used MRI to investigate VWT, cross-sectional area and perimeter in women with and without prolapse, and found no differences [16]. The prolapse group comprised 25 women with prolapse of the vaginal wall or with the cervix at least 2 cm beyond the introitus (grade 3 prolapse). Interestingly in both patients with and without prolapse there was a trend for VWT to increase caudally. This is in accordance with our findings in women with grade 1 or 2 prolapse, but is opposite to our findings in in women with grade 3 prolapse in whom VWT increased towards the introitus. The mean values of VWT acquired were much higher than our values in women with grade 3 prolapse, ranging from 11.6 mm at the introitus to 15.8 mm at the apex; however, the measurements taken (midsagittal diameter) included both the anterior and the posterior vaginal wall. Even taking this into account, the measurements are greater than those found in our study and those seen in histopathological studies [14]. This study was also limited by the fact that measurements were analysed after the images were acquired.

Our findings do not allow comment on the architecture or composition of the vaginal tissue, but morphometric analysis of the smooth muscle component of the vaginal wall has shown a trend for increased thickness in prolapse of grade 3 and above. A study looking at women with posthysterectomy apical vaginal wall prolapse and posterior wall prolapse to the hymen found that in tissue excised from the leading edge of the enterocele, the mean vaginal wall muscularis thickness was 3.5 ± 1.4 mm compared with 2.8 ± 0.9 mm in women without prolapse. The vast majority of prolapse patients in this study had grade 3 and 4 prolapse [9]. Conversely, in a study by Boreham et al., women with prolapse of the posterior vaginal wall demonstrated a decreased fractional area of smooth muscle compared with controls [17], but the authors did not comment on the total VWT. The tissue examined was taken from the apex of the posterior wall, which would correspond, to VWTPF in this study. The fraction of smooth muscle in the muscularis was most diminished in women with grade 3 prolapse, and this finding may correlate with our finding that VWT decreased caudally in women with grade 3 prolapse. The same authors reported similar findings for the anterior vaginal wall [17, 18]. This may be due to increased apoptosis of smooth muscle cells, with studies demonstrating reduced smooth muscle and an increased apoptosis index in women with anterior vaginal wall prolapse compared with women without prolapse [19].

This decrease in smooth muscle can be associated with an increase in the fractional area of connective tissue [20, 21]. In a group of patients with mostly grade 3 prolapse, tissue from the prolapsed anterior wall demonstrated decreased smooth muscle content in the muscularis but increased thickening of the subepithelium/subepethelial collagen fibres [21]. Prolapsed vaginal epithelial tissue has also been found to show reduced total collagen and decreased collagen solubility, but increased collagen turnover [22]. This may explain the thickening we found in tissues beyond the hymen.

In another study comparing vaginal tissue taken from areas of prolapse and tissue taken from nonprolapsed areas, prolapsed tissues demonstrated an increase in collagen III and elastin, as well as increases in smooth muscle cells and collagen cross-linkages (which result in collagens that are brittle and susceptible to rupture) [23]. Collagen III is weaker than collagen I, and this change and is thought to result in thinner collagen fibres with diminished biochemical strength. This change is typical in tissues which are remodelling to adapt to a progressively increasing mechanical load [24]. Elastin is known to modulate the mechanical properties of supportive tissues, and so it has been examined in prolapse. Decreased expression and disordered elastic fibre homeostasis has been attributed to prolapse [25–28]. These findings, along with our own, strengthen the theory of decreased pelvic support leading to prolapse. Interestingly we found no difference in VWT according to menopausal status. This may not be surprising as the literature is conflicting on this topic with some studies showing an increase in VWT in postmenopausal women with prolapse and others showing a decrease [15, 17, 18, 29]. Changes in the vaginal environment including pH, secretions and temperature may also contribute to the changes seen in grade 3 prolapse.

The limitations of our study relate to the lack of body mass index and parity data in our subjects. It was also not possible to blind the sonographer to the grade of prolapse.

## Conclusions

This work adds to the body of work on the aetiology of POP. In women with vaginal prolapse, VWT is less if the prolapse has not descended further than the hymen than if it extends beyond the hymen, and in the latter case VWT is comparable to that in women without prolapse. This may be explained by changes in the vaginal tissue including collagen, elastin and smooth muscle, as well as fibrosis in exposed tissues, rather than by defects in the vagina.
